# Kinsenoside Alleviates Alcoholic Liver Injury by Reducing Oxidative Stress, Inhibiting Endoplasmic Reticulum Stress, and Regulating AMPK-Dependent Autophagy

**DOI:** 10.3389/fphar.2021.747325

**Published:** 2022-01-18

**Authors:** Limin Gao, Xingyu Chen, Zeyu Fu, Jie Yin, Yafen Wang, Weiguang Sun, Hong Ren, Yonghui Zhang

**Affiliations:** ^1^ Biobank, Union Hospital, Tongji Medical College, Huazhong University of Science and Technology, Wuhan, China; ^2^ Department of Clinical Laboratory, the Central Hospital of Wuhan, Wuhan, China; ^3^ Department of Anesthesiology, Union Hospital, Tongji Medical College, Huazhong University of Science and Technology, Wuhan, China; ^4^ Hubei Key Laboratory of Natural Medicinal Chemistry and Resource Evaluation, School of Pharmacy, Tongji Medical College, Huazhong University of Science and Technology, Wuhan, China

**Keywords:** Kinsenoside, alcoholic liver injury, oxidative stress, endoplasmic reticulum stress, AMP-activated protein kinase, autophagy

## Abstract

**Background:** *Anoectochilus roxburghii* (Orchidaceae) is a traditional Chinese medicinal herb with anti-inflammatory, antilipemic, liver protective, immunomodulatory, and other pharmacological activities. Kinsenoside (KD), which shows protective effects against a variety types of liver damage, is an active ingredient extracted from *A. roxburghii*. However, the liver protective effects and potential mechanisms of KD in alcoholic liver disease (ALD) remain unclear. This study aimed to investigate the liver protective activity and potential mechanisms of KD in ALD.

**Methods:** AML12 normal mouse hepatocyte cells were used to detect the protective effect of KD against ethanol-induced cell damage. An alcoholic liver injury model was induced by feeding male C57BL/6J mice with an ethanol-containing liquid diet, in combination with intraperitoneal administration of 5% carbon tetrachloride (CCl_4_) in olive oil. Mice were divided into control, model, silymarin (positive control), and two KD groups, treated with different doses. After treatment, hematoxylin–eosin and Masson’s trichrome staining of liver tissues was performed, and serum alanine aminotransferase (ALT) and aspartate transaminase (AST) levels were determined to assess the protective effect of KD against alcoholic liver injury. Moreover, proteomics techniques were used to explore the potential mechanism of KD action, and ELISA assay, immunohistochemistry, TUNEL assay, and western blotting were used to verify the mechanism.

**Results:** The results showed that KD concentration-dependently reduced ethanol-induced lipid accumulation in AML12 cells. In ALD mice model, the histological examination of liver tissues, combined with the determination of ALT and AST serum levels, demonstrated a protective effect of KD in the alcoholic liver injury mice. In addition, KD treatment markedly enhanced the antioxidant capacity and reduced the endoplasmic reticulum (ER) stress, inflammation, and apoptosis compared with those in the model group. Furthermore, KD increased the phosphorylation level of AMP-activated protein kinase (AMPK), inhibited the mechanistic target of rapamycin, promoted the phosphorylation of ULK1 (Ser555), increased the level of the autophagy marker LC3A/B, and restored ethanol-suppressed autophagic flux, thus activating AMPK-dependent autophagy.

**Conclusion:** This study indicates that KD alleviates alcoholic liver injury by reducing oxidative stress and ER stress, while activating AMPK-dependent autophagy. All results suggested that KD may be a potential therapeutic agent for ALD.

## Introduction

According to the 2016 World Health Organization survey (World Health Organization, 2018), excessive alcohol consumption accounts for 3 million deaths annually (5.3% of all deaths). Alcoholic liver disease (ALD) is a chronic disease caused by long-term heavy drinking and includes steatosis, steatohepatitis, fibrosis, cirrhosis, and hepatocarcinoma (Kong et al., 2019). Among long-term (months or years) heavy (>40 g of alcohol per day) drinkers, 90–100% develop alcoholic fatty liver, and 10–35% of them develop alcoholic steatohepatitis. Furthermore, 8–20% of patients with alcoholic steatohepatitis develop liver cirrhosis, and 2% of patients with liver cirrhosis progress to hepatocarcinoma (Seitz et al., 2018). ALD seriously affects human’s health and quality of life.

The early stage of ALD is alcoholic fatty liver, caused by abnormal lipid accumulation. Autophagy is the mechanism of self-protection of cells under stress, which can prevent lipid accumulation by degrading lipids (You et al., 2018) and can also prevent cell damage (Dikic and Elazar, 2018). However, chronic alcohol drinking inhibits autophagy, which is one of the causes of lipid metabolism disorders (Chao et al., 2018). AMP-activated protein kinase (AMPK) is an important regulator of various metabolic and signal transduction pathways, including autophagy (Xiang et al., 2020). Ethanol can inhibit AMPK, induce fatty acid synthesis, inhibit fatty acid oxidation, and cause abnormal fat accumulation in the liver (You et al., 2004). Continuous drinking of alcohol can aggravate liver damage in patients with fatty liver. The damage can result in the transformation of hepatic stellate cells, which are normally in a resting state, into activated myofibroblasts, lead to the upregulation of α-smooth muscle actin and the deposition of the extracellular matrix, and cause liver fibrosis, a severe form of liver injury (Seitz et al., 2018). In addition, acetaldehyde and reactive oxygen species (ROS), the metabolites of ethanol, can trigger oxidative stress and endoplasmic reticulum (ER) stress in hepatocytes, which in turn triggers inflammation and apoptosis, also leading to liver injury (Dara et al., 2011; Ceni et al., 2014). Fibrosis is a critical pathological process in ALD, but recent studies have shown that fibrosis can be reversed with a timely intervention (Parola and Pinzani, 2019).

The clinical treatment for ALD mainly consists of antioxidant and anti-inflammatory drugs, such as silymarin and glycyrrhizic acid preparations; however, these drugs have limited curative effects and a limited therapeutic scope (Flora et al., 1998; Levy et al., 2004; Meng et al., 2018; Li X. et al., 2019). Thus, it is essential to develop novel, safe, and pathophysiology-oriented therapies for ALD.

**FIGURE 1:**
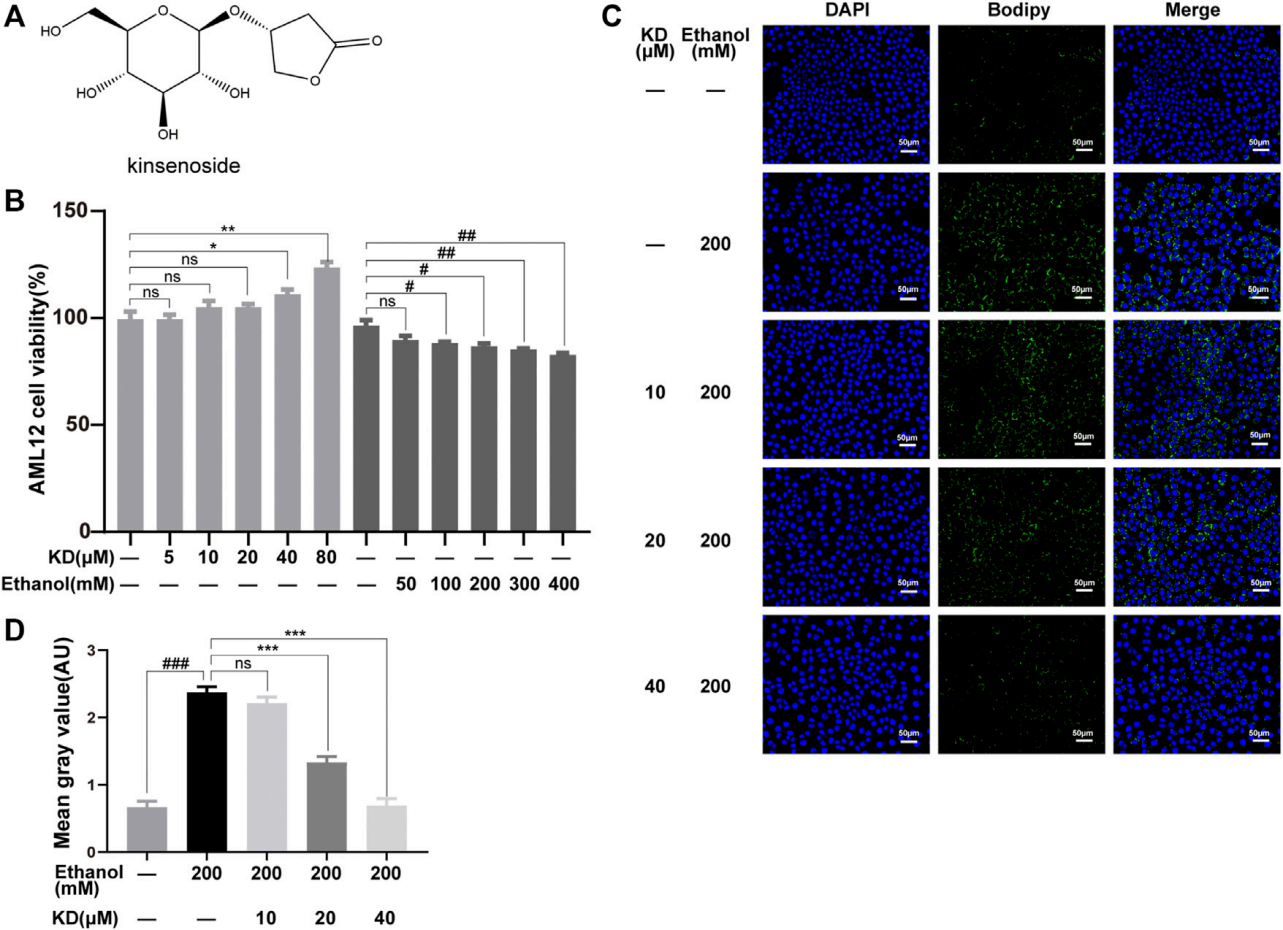
Effects of KD on cellular ethanol-induced lipid accumulation. **(A)** The chemical structure of KD. **(B)** Effects of KD and ethanol on AML12 cell viability (*n* = 4). **(C)** Images of BODIPY-stained AML12 cells treated with KD and/or ethanol for 48 h. Scale bar: 50 µm. **(D)** Quantitative analysis of BODIPY staining (*n* = 3). Data represent the mean ± SEM. ns, not significant, *p < 0.05, **p < 0.01, ***p < 0.001, KD-treated vs. untreated cells. ^#^ *p* < 0.05, ^##^ *p* < 0.01, ^###^ *p* < 0.001, alcohol-treated vs. untreated cells.

*Anoectochilus roxburghii* (Wall.) Lindl. (Orchidaceae), also known as Jinxianlan (Simplified Chinese: 金线兰) and Jinxianlian (Simplified Chinese: 金线莲), is a perennial herb that is mainly grown in China, Japan, India, Nepal, and Sri Lanka. In China, *A. roxburghii* is a protected species and is used as a traditional Chinese medicinal material for the treatment of liver disease, diabetes, hypertension, cancers, hand-foot-and-mouth disease, and osteoarthritis (Ye et al., 2017; Tang et al., 2018; Guo et al., 2019; Liu Y. et al., 2020). Kinsenoside (KD), 3-*O*-β-D-glucopyranosyl-(3*R*)-hydroxybutanolide (Figure 1A), a major active ingredient extracted from *A. roxburghii*, exhibits anti-inflammatory, antilipemic, liver protective, immunomodulatory, and other pharmacological activities (Qi et al., 2018; Wang et al., 2019; Zhou et al., 2019). Our previous reports have demonstrated that KD treatment could alleviate the complications of 17α-ethinylestradiol-induced cholestatic liver injury and concanavalin A-induced autoimmune liver injury by inhibiting inflammatory responses and regulating immune balance (Xiang et al., 2016; Ming et al., 2021). However, it is unclear whether KD has therapeutic activity against alcohol-induced liver injury. In this study, we aimed to investigate the protective effect and the underlying mechanism of KD against ALD.

## Materials and Methods

### Materials

A whole herb of *A. roxburghii* was purchased from Yongan City Huangnijia Co., Ltd. (Sanming, China) and identified by Prof. Jianping Wang at the School of Pharmacy, Huazhong University of Science and Technology. A voucher specimen (No. TJ20180901) has been deposited at the Hubei Key Laboratory of Natural Medicinal Chemistry and Resource Evaluation. KD was prepared using a previously reported method (Ming et al., 2021). The purity of KD (>98%) was analyzed using a high-pressure liquid chromatograph equipped with an evaporative light scattering detector, and the identity was confirmed by comparing its nuclear magnetic resonance data with those from a previous study (Xiang et al., 2016). Silymarin was purchased from Sigma Chemical Co. (St. Louis, MO, United States). The Lieber–DeCarli ethanol liquid diet was purchased from Trophic Animal Feed High-tech Co., Ltd. (Jiangsu, China). 3-Nitrotyrosine (3-NT; ab61392), 4-hydroxynonenal (4-HNE; ab46545), catalase (ab76110), aldehyde dehydrogenase 2 (ALDH2; ab108306), glyceraldehyde 3-phosphate dehydrogenase (GAPDH; ab181602), cluster of differentiation 3 (CD3; ab215212), Bcl-XL (ab32370), Bax (ab32503), STRAD (ab192879) and phospho (p)-mTOR (ab109268) antibodies were purchased from Abcam (Cambridge, United Kingdom). AMPKα (5832), p-AMPKα (2535), Beclin-1 (3495), p-Beclin-1 (84966S), BIP (3177), eIF2α (5324), p-eIF2α (3398), F4/80 (70076), JNK1 (3708), p-SAPK/JNK (4668), LC3A/B (12741), Raptor (2280), p-Raptor (2083), sequestosome-1 (SQSTM1)/p62 (5114), ULK1 (8054), p-ULK1 (5869), mTOR (2983), goat anti-rabbit (5151), and goat anti-mouse (5257) antibodies were purchased from Cell Signaling Technology (Beverly, MA, United States). Alcohol dehydrogenase 1B (ADH1B; 66939-1-Ig), LKB1 (10746-1-AP) and cytochrome P450 2E1 (CYP2E1; 19937-1-AP) antibodies were purchased from Proteintech (Rosemount, IL, United States). The CHOP (WL00880) antibody was purchased from Wanleibio (Liaoning, China). The mCherry-EGFP-LC3 adenovirus was purchased from Hanbio Co. Ltd. (Hanbio, Shanghai, China).

### Cell Culture

The mouse hepatocyte cell line, AML12, was cultured in Dulbecco’s modified Eagle’s medium/F12 (Zhong Qiao Xin Zhou, China) supplemented with 10% fetal bovine serum, 1% penicillin–streptomycin, 10 μg/ml insulin, 5.5 μg/ml transferrin, 5 ng/ml selenium, and 40 ng/ml dexamethasone. Cells were cultured in a humidified atmosphere containing 5% CO_2_ at 37°C.

### Cell Viability

AML12 cells at the third passage were seeded into 96-well plates at a density of 1 × 10^4^ cells per well. After 24 h of incubation, the cells were washed with phosphate-buffered saline (PBS) and then cultured with different concentrations of KD (0, 5, 10, 20, 40, and 80 μM) or ethanol (0, 50, 100, 200, 300, and 400 mM). After incubation for another 24 h, the culture media were replaced with a culture medium containing a 10% Cell Counting Kit-8 solution. After incubation for 2 h, the absorbance was measured at 450 nm (Varioskan LUX spectrophotometer; Thermo Fisher Scientific, Inc.).

### BODIPY Staining

AML12 cells at the fifth passage were seeded at a density of 2 × 10^5^ cell per well into a 6-well plate with slides. On the next day, the medium was changed, and the cells were incubated with KD (0, 10, 20, and 40 μM) for 1 h, followed by incubation with or without 200 mM ethanol for 24 h, after which medium containing KD or ethanol was replaced. After another 24 h, the cells were washed with PBS, fixed with paraformaldehyde, and then stained with the BODIPY staining solution (B&P Biotech, Hangzhou, China) according to the manufacturer’s instructions. 4′,6-Diamidino-2-phenylindole (Servicebio, Wuhan, China) was used to counterstain the nuclei. Images were observed under a fluorescence microscope (Olympus, Tokyo, Japan). The ImageJ software was used for quantitative analysis of lipids.

### Animal Experiments

The protocol of the animal study was reviewed and approved by the Institutional Animal Care and Use Committee of the Tongji Medical College, Huazhong University of Science and Technology. The Lieber–DeCarli ethanol liquid diet was used according to the manufacturer’s instructions and was combined with carbon tetrachloride (CCl_4_) to induce alcoholic liver injury in mice (Lamas-Paz et al., 2018). Eight-week-old male C57BL/6J mice (21–23 g) were purchased from SPF Biotechnology Co., Ltd. (Beijing, China) and were raised in a specific pathogen-free environment. Silymarin was chosen as the positive control (Federico et al., 2017; Gillessen and Schmidt, 2020). All mice were first fed a control (ethanol-free) liquid diet for 5 days. Subsequently, the mice were randomly divided into the following five groups: control, model, KD-20 mg/kg, KD-40 mg/kg, and silymarin-80 mg/kg. The control group was administered the control liquid feed, while the other groups were administered the ethanol liquid feed, and the ethanol concentration was gradually increased to 4% (w/v) in the first week and then maintained at the same level. On week 5, the mice in all groups, except the control group, were intraperitoneally injected with 5% CCl_4_ in olive oil (2 ml/kg) twice a week and continued in subsequent weeks. The mice were sacrificed 5 weeks after the first injection of CCl_4_ to obtain tissues and blood.

### Tandem Mass Tag Quantitative Proteomics

Samples were lysed with a buffer containing 4% sodium dodecyl sulfate, 100 mM Tris-HCl, and 1 mM dithiothreitol (pH 7.6) to extract proteins, and the bicinchoninic acid method was used for protein quantification. An appropriate amount of protein from each sample was prepared using the filter-aided sample preparation method for trypsin digestion (Wisniewski et al., 2009). An Empore™ solid phase extraction cartridge (C18, standard density, bed internal diameter: 7 mm, volume: 3 ml; Sigma) was used to desalt the peptides. The peptides were concentrated by vacuum centrifugation and then reconstituted with 40 μL of 0.1% (v/v) formic acid. Peptides (100 μg) from each sample were labeled according to the manufacturer’s instructions (Thermo Fisher Scientific) and then fractionated using a high pH reversed-phase peptide fractionation kit (Thermo Fisher Scientific). Liquid chromatography–tandem mass spectrometry was performed on a Q Exactive mass spectrometer (Thermo Fisher Scientific) coupled with an EASY-nLC liquid chromatograph (Thermo Fisher Scientific) to analyze the peptides. Protein identification and quantitative analyses were performed by searching the mass spectrometric raw data of each sample using the MASCOT search engine version 2.2 (Matrix Science, London, United Kingdom) integrated into the Proteome Discoverer 1.4 software. Finally, bioinformatics analysis was performed as previously described (Ashburner et al., 2000; Ross et al., 2004; Gotz et al., 2008).

### Measurement of Serum and Liver Biochemical Parameters

Serum levels of tumor necrosis factor-alpha (TNF-α) and interleukin-6 (IL-6) were measured using an enzyme-linked immunosorbent assay kits (Neobioscience) according to the manufacturer’s instructions. Serum levels of alanine aminotransferase (ALT) and aspartate transaminase (AST), as well as triglycerides (TG), reduced glutathione/oxidized glutathione (GSH/GSSG) and nicotinamide adenine dinucleotide (NAD^+^)/reduced NAD^+^ (NAD^+^/NADH) ratios of liver homogenate, were determined according to the instructions of each kit obtained from Nanjing Jiancheng Bioengineering Institute. Activities of ADH, catalase, ALDH, and superoxide dismutase (SOD) in liver homogenates were also measured according to the instructions of each kit obtained from Nanjing Jiancheng Bioengineering Institute.

### Histopathology

Liver tissue was fixed with 10% formalin and embedded in paraffin. The paraffinized tissue was cut into 5-μm sections and subjected to hematoxylin–eosin (H&E) and Masson’s trichrome staining.

### Immunohistochemistry

The levels of 3-NT, 4-HNE, F4/80, and CD3 in the liver were determined by immunohistochemical staining. Paraffin sections of the liver tissue were prepared using high temperature and pressure and then incubated with 3% hydrogen peroxide for 20 min to inactivate endogenous peroxidase. After incubation with 10% serum for 20 min to block nonspecific binding, the sections were separately incubated with the 3-NT, 4-HNE, F4/80, and CD3 primary antibodies in a working solution, followed by incubation with the corresponding secondary antibody to develop color. After the slides were rinsed with Tris-buffered saline, 3,3′-diaminobenzidine was added, and the sections were observed under a microscope. Subsequently, the slides were rinsed with tap water, counterstained with hematoxylin, dehydrated with gradient ethanol, and finally loaded with a neutral adhesive for microscopic examination.

### Terminal Deoxynucleotidyl Transferase dUTP Nick-End Labeling Assay

A TUNEL kit (Roche, Shanghai, China) was used to detect apoptosis in liver tissue. Paraffin sections were deparaffinized and hydrated. A mixture of 3% bovine serum albumin and 20% normal calf serum was used to block nonspecific reactions. The TUNEL working solution was added dropwise, and the sections were incubated in the dark for 60 min at 37°C. 4′,6-Diamidino-2-phenylindole was used to counterstain the nuclei, and images were observed under a fluorescence microscope.

### Western Blotting

The liver tissue was removed from storage at −80°C and lysed in a buffer containing protease and phosphatase inhibitors. The lysate was then centrifuged at 12,000 × *g* for 10 min, and the protein concentration was quantified in the supernatant using the bicinchoninic acid method. After boiling with a sample loading buffer for 5 min, approximately 20–50 µg of total protein was separated by sodium dodecyl sulfate–polyacrylamide gel electrophoresis. The separated proteins were transferred onto nitrocellulose membranes, and the membranes were blocked with a fast blocking solution, followed by incubation with primary antibodies overnight at 4°C. After washing with Tris-buffered saline with 0.1% Tween 20, the membranes were incubated with secondary antibodies for 30–60 min at room temperature. A dual-color near-infrared fluorescence imaging system was used to detect protein bands. The ImageJ software was used for quantitative analysis of proteins.

### Autophagy Progress in Ethanol Treated AML12 Cells

1 × 10^6^ AML12 cells were seeded in a 6-well plate. The next day, the cells were treated with 100 mM ethanol for 0, 1, 4, 8, 12, and 24 h. After the cells were collected, western blotting was performed to detect the expression levels of LC3A/B-I, LC3A/B-II and SQSTM1.

The AML12 cells were seeded into 6-well plates at a density of 1 × 10^6^ cells/well for 24 h. Then, 40 μM KD with or without 50 μM chloroquine (CQ) were added for 1 h according to the grouping, and cells were treated with 100 mM ethanol for another 8 h. The expression levels of LC3A/B-I, LC3A/B-II and SQSTM1 were further detected as described above.

### Confocal Microscopy Assay

The AML12 cells were seeded in a confocal culture dish with a glass bottom (diameter of 15 mm; 1.5 × 10^5^ cells/dish, and transfection was carried out on the next day. After 39 h of transfection, 40 μM KD or 50 μM CQ was added for 1 h, followed by addition of 100 mM ethanol for 8 h. After being washed with PBS, the cells were fixed with 4% paraformaldehyde for 10 min, and then washed with PBS again. The cells were immersed in PBS for observation by confocal fluorescence (Nikon, Tokyo, Japan).

### Statistical Analysis

Data are presented as the mean ± standard error of the mean (SEM) and were analyzed with Dunnett’s *t*-test or one-way analysis of variance using the GraphPad Prism software (GraphPad Software, Inc., San Diego, CA, United States). Statistical significance was set at *p* < 0.05.

## Results

### Kinsenoside Reduces Ethanol-Induced Lipid Accumulation in Hepatocytes

AML12 normal hepatocytes were selected to analyze the effect of KD on ethanol-induced lipid accumulation. The cytotoxicity of KD and ethanol to AML12 cells was assayed to determine the optimal concentrations for subsequent experiments. As shown in Figure 1B, KD showed no cytotoxic effect on AML12 cells at 80 μM, while ethanol caused liver cell damage in a concentration-dependent manner in a range of 100–400 mM. Based on the above results, 200 mM ethanol was chosen for further treatment. BODIPY staining was used to localize lipids, which showed that 200 mM ethanol induced obvious lipid accumulation (green fluorescence intensity in Figure 1C). However, after pretreatment with different concentrations of KD, the lipid accumulation levels significantly decreased, and the lipid accumulation status was comparable to that of normal cells (Figure 1C). The BODIPY staining results showed that KD reduced ethanol-induced lipid accumulation in a concentration-dependent manner in a range from 20 to 40 μM (Figure 1D).

### Kinsenoside Alleviates Alcoholic Liver Injury in Mice

**FIGURE 2:**
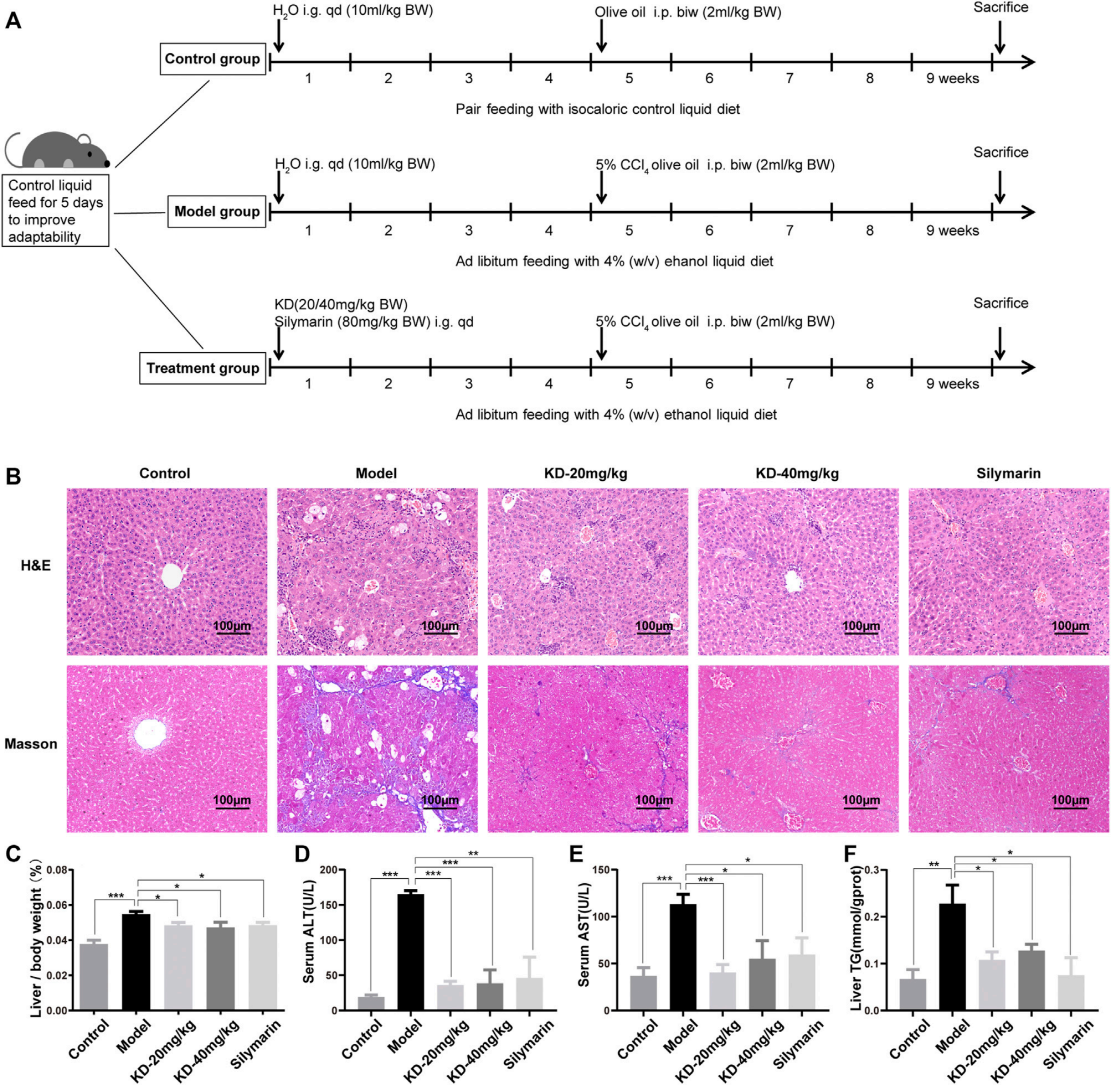
Effects of KD on alcoholic liver injury in mice. **(A)** Schematic representation of the modeling of alcoholic liver injury in mice. **(B)** Images of H&E and Masson’s trichrome-stained sections of the mice liver. Scale bar: 100 µm. **(C)** Liver to body weight ratio. **(D)** ALT levels in the mice serum. **(E)** AST levels in the mice serum. **(F)** TG content of mice liver homogenate. (*n* = 5–7). Data represent the mean ± SEM. *p < 0.05, **p < 0.01, ***p < 0.001 vs. the model group.

A mouse model was established and pathologically analyzed to explore whether KD alleviates alcoholic liver injury. The detailed modeling method is illustrated in Figure 2A. The results of H&E staining showed ballooning degeneration and inflammatory cell infiltration in the model group. After KD treatment, ballooning degeneration was significantly reduced, and inflammation was alleviated (Figure 2B). Masson’s trichrome staining showed that the number of blue collagen fibers dramatically increased in the model group and decreased after KD treatment, indicating the therapeutic effect of KD against liver tissue fibrosis (Figure 2B). The liver index of mice also changed after ethanol and KD treatment. KD reduced the increase in liver to body weight ratio caused by ethanol (Figure 2C) The essential indicators of hepatocellular damage were further assessed, including ALT, AST and TG. As shown in Figures 2D–F, the ALT, AST and TG levels were obviously elevated in the model group compared with those in the control group. KD at 20 and 40 mg/kg significantly (*p* < 0.05) decreased the levels of these biomarkers. These findings demonstrated that KD alleviated alcoholic liver injury in mice, and its effects were comparable to those of silymarin.

### Proteomic Analysis of the Mechanism of Action of Kinsenoside in Mice

**FIGURE 3:**
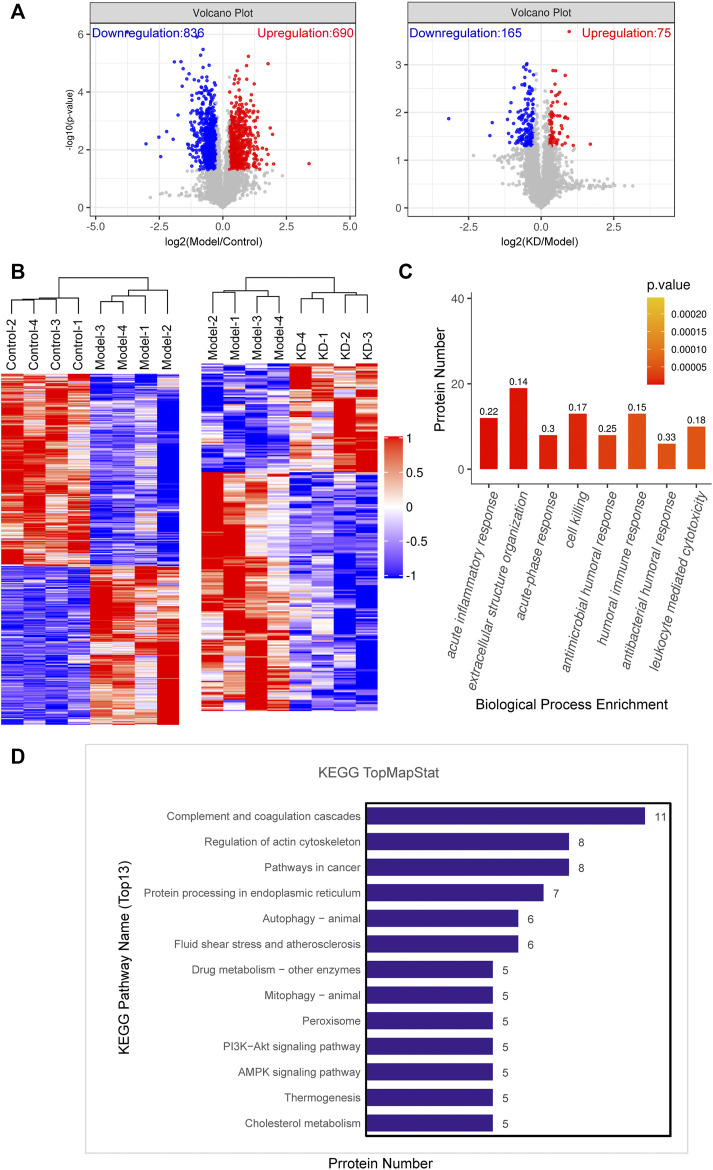
Data from proteomic analysis of mice liver tissue. **(A)** Volcano plots of the number of differentially expressed proteins between the model and control groups and the KD and model groups. **(B)** Cluster analysis of the differentially expressed proteins in the control, model, and KD-treated groups of mice. **(C)** Biological process classification of GO functional enrichment histogram between the KD and model groups. The numbers on the histogram represent rich factor. The rich factor represents the ratio of the number of differentially expressed proteins annotated to a GO functional category to the number of all identified proteins annotated to the GO functional category. **(D)** KEGG pathway annotation of the top 13 differentially expressed proteins between the KD and model groups. (*n* = 4).

To explore the mechanism of action of KD in alleviating alcoholic liver injury, proteomics was used for screening. The proteomic volcano plots and heatmap show that protein expression significantly differed between the model and control groups, with 836 proteins downregulated and 690 proteins upregulated in the model group. Compared with their expression in the model group, 165 proteins were downregulated and 75 proteins were upregulated in the KD group (Figure 3A). Cluster analysis of differentially expressed proteins showed that the protein expression levels in the liver tissue after KD treatment were significantly different from that in model group (Figure 3B). Gene Ontology (GO) analysis of the differentially expressed proteins between the KD and model groups showed that the enriched biological process terms were mainly associated with acute inflammatory response, extracellular structure organization, acute-phase response and cell killing (Figure 3C). Kyoto Encyclopedia of Genes and Genomes (KEGG) pathway enrichment analysis showed that differentially expressed proteins between the KD and model groups (Figure 3D), in combination with the pathogenesis of ALD, were mainly involved in the ER, autophagy, and AMPK signaling pathways.

### Kinsenoside Alleviates Oxidative Stress and ER Stress in Mice

**FIGURE 4:**
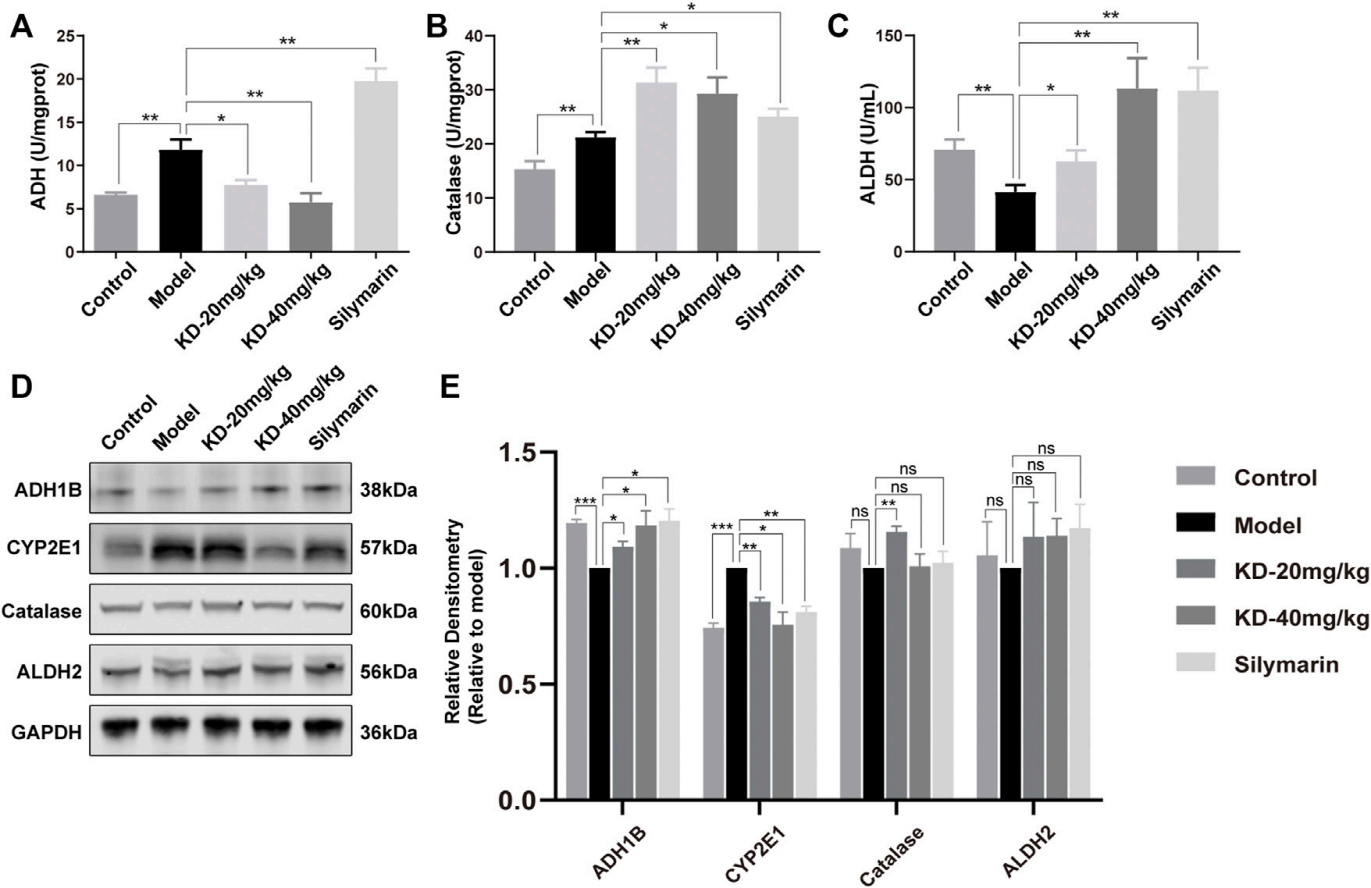
Effects of KD on ethanol metabolism in mice. **(A)** ADH activity in mice liver homogenates. **(B)** Catalase activity in mice liver homogenates. **(C)** ALDH activity in mice liver homogenates. (*n* = 5–7). **(D)** Expression of the ADH1B, catalase, CYP2E1, and ALDH2 proteins in mice liver tissue. **(E)** Quantitative analysis of protein expression (*n* = 3). GAPDH was used as a loading control and for data normalization. Data represent the mean ± SEM. ns, not significant. *p < 0.05, **p < 0.01, ***p < 0.001 vs. the model group.

The metabolism of ethanol *in vivo* is an important physiological and pathological process in ALD development. After entering the body, ethanol is converted into acetaldehyde by ADH, CYP2E1 and catalase. Acetaldehyde is then converted into nontoxic acetic acid by ALDH (Seitz et al., 2018). Analysis of enzymes of ethanol metabolism in the liver of mice showed that KD inhibited the ethanol-induced increase in ADH activity, enhanced the increase in catalase activity, and reversed the decrease in ALDH activity (Figures 4A–C). Western blotting revealed that KD reversed the ethanol-induced decrease in ADH1B (the main subunit of ADH) expression and the increase in CYP2E1 expression but had no effect on the expression of catalase and ALDH2 (the main subunit of ALDH) (Figures 4D,E).

**FIGURE 5:**
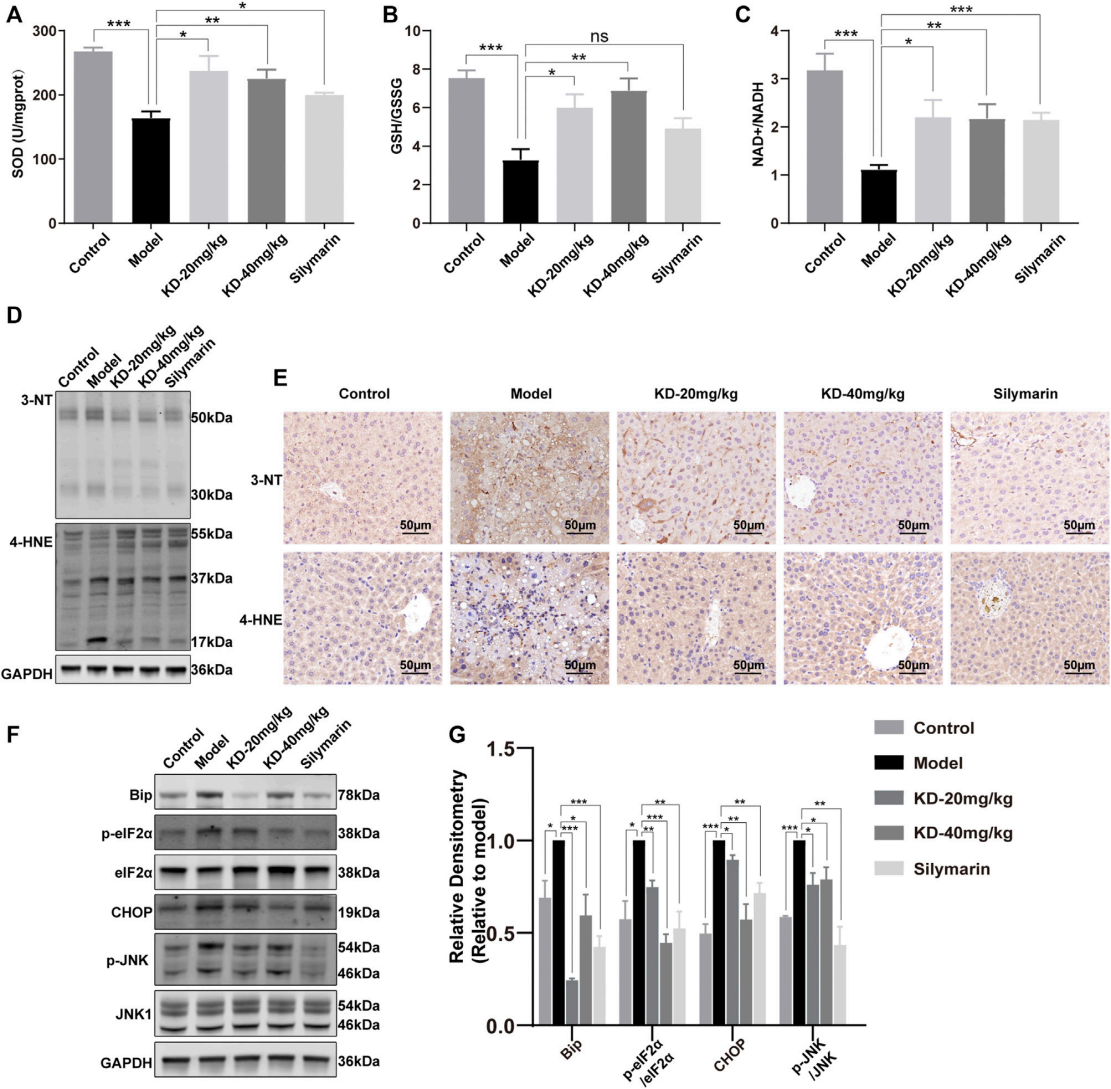
Effects of KD on oxidative stress and ER stress in mice. **(A)** SOD activity in liver homogenates. **(B)** Liver GSH to GSSG ratios. **(C)** Liver NAD^+^ to NADH ratios. (*n* = 5–7). **(D)** Liver 3-NT and 4-HNE expression determined by western blotting. **(E)** Immunohistochemistry staining of 3-NT and 4-HNE in the mice liver. Scale bar: 50 µm. **(F)** Expression of ER stress-related proteins determined by western blotting. **(G)** Quantitative analysis of protein expression (*n* = 3). GAPDH was used as a loading control. Data represent the mean ± SEM. ns, not significant. *p < 0.05, **p < 0.01, ***p < 0.001 vs. the model group.

ROS, another byproduct of ethanol metabolism, can cause oxidative stress, together with acetaldehyde (Farfan Labonne et al., 2009; Seitz et al., 2018). Analysis of the oxidative stress indicators SOD, GSH/GSSG, and NAD^+^/NADH showed that KD improved the antioxidant status in mice (Figures 5A–C). In the presence of ROS, reactive nitrogen species are derived from NO and nitrify tyrosine or protein tyrosine residues to produce 3-NT. The 3-NT level in the diseased state is significantly higher than the normal 3-NT level (Li Y. et al., 2019). 4-HNE is an aldehyde product of lipid peroxidation and is considered a key mediator of oxidative stress-caused cell death (Ma et al., 2020; Hyatt et al., 2021). The ethanol-induced increases in the 3-NT and 4-HNE levels were reversed by KD treatment (Figures 5D,E). These results indicated that KD reduced the ethanol-induced oxidative stress.

ROS and acetaldehyde cause not only oxidative stress but also ER stress by damaging proteins and DNA (Ji, 2012; Ji, 2014; Ganne-Carrié and Nahon, 2019). KEGG analysis of proteomics data also showed the association of differentially expressed proteins with ER processes (Figure 3D). Western blotting showed that KD reduced the levels of the key ER stress protein BIP and the transcription factor CHOP, as well as the phosphorylation of eIF2α, thereby alleviating the translation block. JNK, a key kinase in ER stress, was also suppressed (Figures 5F,G). These results suggested that KD inhibited the persistent ER stress caused by long-term ethanol consumption.

**FIGURE 6:**
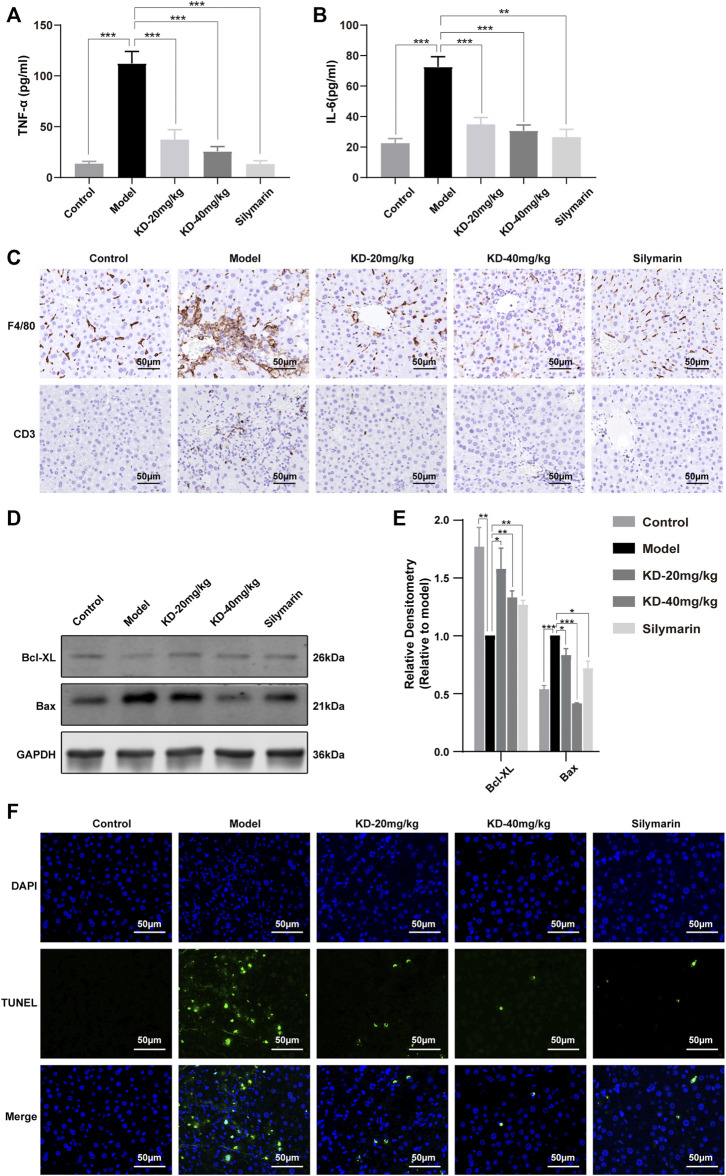
Effects of KD on ethanol-induced liver inflammation and hepatocyte apoptosis in mice. **(A)** Serum TNF-α levels. **(B)** Serum IL-6 levels. (*n* = 5–7). **(C)** Immunohistochemistry staining for F4/80 and CD3 in the mice liver. **(D)** Expression of apoptosis-related proteins determined by western blotting. **(E)** Quantitative analysis of protein expression (*n* = 3). GAPDH was used as a loading control. **(F)** TUNEL-stained liver sections. Scale bar: 50 µm. Data represent the mean ± SEM. *p < 0.05, **p < 0.01, ***p < 0.001 vs. the model group.

Oxidative stress and ER stress can trigger inflammation and cell apoptosis. Our results showed that KD reversed the liver inflammation-associated increases in the levels of serum TNF-α and IL-6 (Figures 6A,B). Immunohistochemistry staining of liver tissue sections showed that the ethanol-induced increases in the F4/80 and CD3 levels were reversed by KD (Figure 6C). In addition, CHOP and JNK activation, which can trigger cell apoptosis, was inhibited, while the expression of the antiapoptotic factor Bcl-XL was increased and that of the proapoptotic factor Bax was decreased by KD treatment (Figures 6D,E). The TUNEL assay also confirmed that the ethanol-induced apoptosis of hepatocytes decreased after KD administration (Figure 6F). Thus, the results showed that KD inhibited the ethanol-induced liver inflammation and cell apoptosis. Together, KD alleviates oxidative stress and ER stress by affecting ethanol metabolism in the liver, thereby reducing inflammation and cell apoptosis, which in turn relieves alcoholic liver injury.

### Kinsenoside Activates AMPK-Dependent Protective Autophagy in Mice With Alcoholic Liver Injury

**FIGURE 7:**
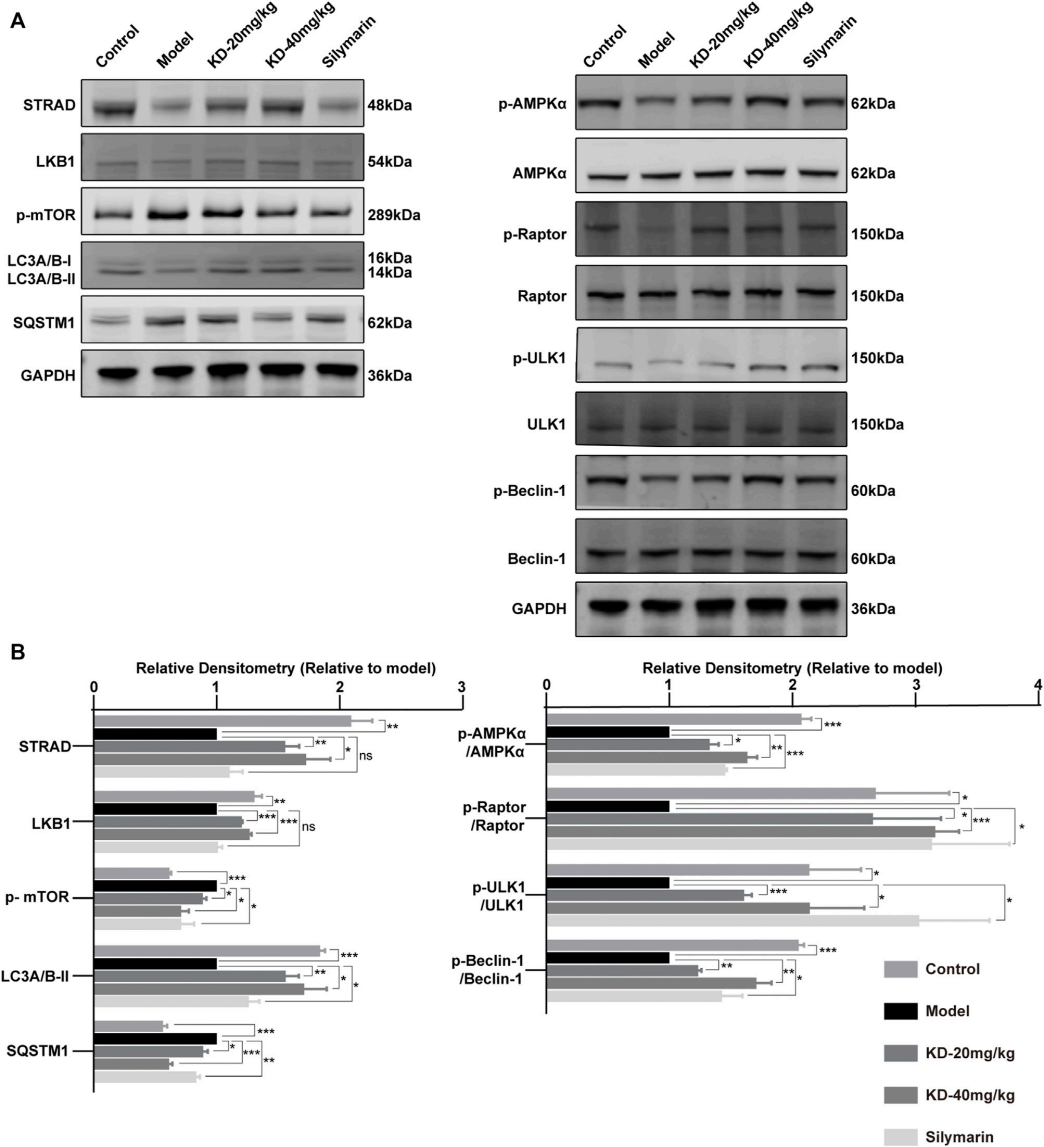
Activation of AMPK-dependent protective autophagy in KD-treated mice with alcoholic liver injury. **(A)** Levels of AMPK and autophagy-related proteins determined by western blotting. **(B)** Quantitative analysis of protein expression (*n* = 3). Data represent the mean ± SEM. GAPDH was used as a loading control. ns, not significant. *p < 0.05, **p < 0.01, ***p < 0.001 vs. the model group.

AMPK, a central regulator of eukaryotic cell and organism metabolism, is a key protein involved in a variety of signaling pathways. Long-term drinking inhibits the activity of AMPK, thereby disrupting metabolic processes (Gao and Bataller, 2011; Seitz et al., 2018). Our results showed that KD increased the protein levels of STRAD and LKB1 (Figures 7A,B), which were the upstream of AMPK, thereby promoting the phosphorylation of AMPK.

Autophagy can remove damaged proteins, organelles, and lipids from cells and maintain intracellular balance. The increases in ROS and acetaldehyde levels caused by long-term alcohol drinking can destroy proteins, cause lipid accumulation, and inhibit liver autophagy, thereby aggravating the alcohol-induced liver injury (Wang et al., 2015; Allaire et al., 2019). Our results showed that KD treatment led to the phosphorylation of Raptor by activated AMPK, thereby directly inhibiting the phosphorylation of mTOR (Total mTOR strip is shown in Supplementary Figure S1). MTOR is an autophagy inhibitory protein, and the inhibition of mTOR by AMPK can activate autophagy (Allaire et al., 2019). In addition, the activated AMPK in the KD group could directly activate autophagy *via* phosphorylation of ULK1 at Ser555. KD treatment increased the phosphorylation level of Beclin-1, a key autophagy protein, and that of LC3A/B-II and decreased the level of the autophagy substrate SQSTM1 (Turco et al., 2019), indicating enhanced autophagy (Galluzzi and Green, 2019). The results suggested that KD reversed the ethanol-induced inhibition of the AMPK signaling pathway and autophagy (Figure 7A,B). In summary, in mice with alcoholic liver injury, KD treatment led to the upregulation of STRAD/LKB1, resulting in the activation of AMPK-dependent protective autophagy; which was also shown in Supplementary Figure S2 that the protective effect of KD was abrogated by autophagy inhibition. Silymarin also enhanced protective autophagy by promoting AMPK phosphorylation but had no influence on STRAD and LKB1 expression.

### Kinsenoside Restores Ethanol-Suppressed Autophagic Flux

**FIGURE 8:**
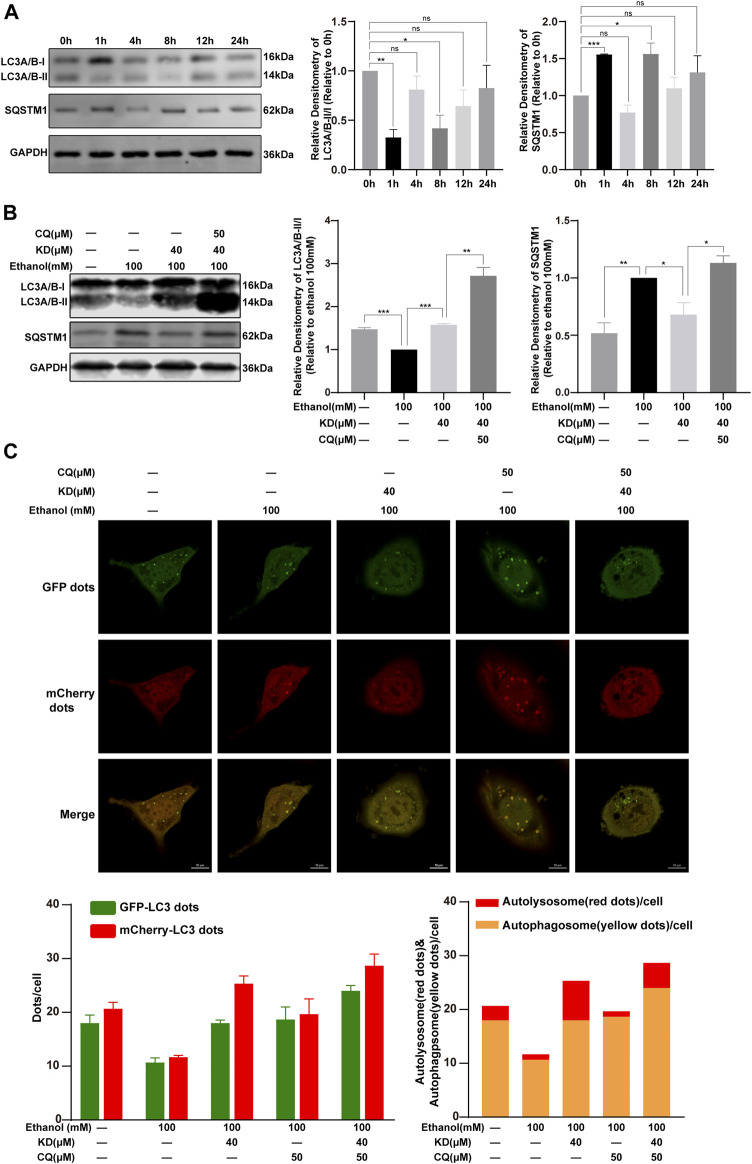
KD restores ethanol-suppressed autophagic flux. **(A)** The effect of 100 mM ethanol for different time on autophagy. **(B)** KD affect the autophagy of ethanol in cells. **(C)** Confocal fluorescence image and analysis of KD promoting the recovery of autophagic flux. Red puncta represent autolysosomes, yellow puncta (merged by red and green) represent autophagosomes. (*n* = 3). Data represent the mean ± SEM. GAPDH was used as a loading control. ns, not significant. *p < 0.05, **p < 0.01, ***p < 0.001. Scale bar: 10 µm.

In order to determine the role of KD in autophagy of alcoholic liver injury, we exposed AML12 cells to 100 mM ethanol for 0, 1, 4, 8, 12, and 24 h. The results of multiple experiments demonstrated that the phenomenon of autophagy was a dynamic process and ethanol inhibited LC3A/B-II expression at 8 h with higher stability (Figure 8A), we thus chose 8 h as the end point to study the changes of autophagic flux.

Cells treat with 100 mM ethanol for 8 h showed reduced expression of LC3A/B-II and increased expression of SQSTM1, indicating that autophagy was inhibited. This change was reversed after KD treatment, which means that KD could activated the suppressed autophagy induced by 100 mM ethanol. LC3A/B-II and SQSTM1 both increased after adding KD and autophagy inhibitors CQ, revealing that KD had no effect on autophagy inhibited by CQ (Figure 8B).

For further proof, the AML12 cells were transfected with mCherry-EGFP-LC3 adenovirus. Because the EGFP fluorescence is quenched in the acidic environment of the lysosome, the EGFP and mCherry fluorescence express at the same time, which means the yellow dots after the merge indicate the autophagosomes. Fluorescent dots that do not express EGFP but express mCherry indicate autolysosomes (Liu B. et al., 2020). It was found that 100 mM ethanol reduced the number of autophagosomes and autolysosomes. KD treatment increased the number of autophagosomes and autolysosomes. Treatment with CQ inhibited the binding of autophagosomes and lysosomes, and further caused the accumulation of autophagosomes. After the cells treated with KD and CQ at the same time, the number of autophagosomes was increased, meanwhile the increase ratio of autolysosomes was less than that of KD alone. In summary, the data suggested that KD increases the production of autophagosomes, and could partially restore the autolysosome function inhibited by 100 mM ethanol, thereby enhancing autophagic flux (Figure 8C).

## Discussion

Chronic alcohol use can result in liver injury, which further leads to fibrosis, cirrhosis, and even liver cancer. Early treatment is critical to prevent liver disease progression. It has been reported that KD possesses broad pharmacological activities, including hepatoprotective, antihyperglycemic, anti-inflammatory, and antiosteoporosis effects (Qi et al., 2018).

In this study, the *in vitro* results showed that KD was nontoxic to AML12 cells and reduced ethanol-induced lipid accumulation in a concentration-dependent manner. The early stage of ALD is presented as abnormal lipid accumulation, and long-term chronic drinking can lead to alcoholic fatty liver, alcoholic steatohepatitis, alcoholic liver fibrosis, cirrhosis, and liver cancer (Seitz et al., 2018). Liver fibrosis is the most serious but curable form of ALD. Therefore, for *in vivo* studies, alcoholic liver fibrosis was chosen as the endpoint of liver injury. Many studies have shown that it is difficult to simulate severe liver injury in mice using ethanol alone, and the assistance of a second hit is required (Lamas-Paz et al., 2018; Brol et al., 2019). Therefore, in the current study, a combination of ethanol and CCl_4_ was used to induce severe liver injury (Satishchandran et al., 2018; Brol et al., 2019). We found that KD had a protective effect in this model, as evidenced by the reduction of serum aminopherases, liver inflammation, and the degree of fibrosis.

Because of the reduction of NAD^+^ to NADH by ADH and ALDH2, ethanol metabolism results in a decrease in the NAD^+^/NADH ratio, and NADH is considered the main source of ROS in mitochondria (Ying, 2008). Moreover, previous studies have shown that long-term alcohol consumption induces CYP2E1 (Zhong et al., 2015). CYP2E1 metabolizes ethanol and produces ROS (Abdelmegeed et al., 2013), which, together with acetaldehyde, damage proteins and DNA. In this study, analysis of enzymes of ethanol metabolism in the liver showed that KD treatment increased ADH1B expression, enhanced the activity of ALDH2, reduced CYP2E1 expression, and reduced the accumulation of ROS and acetaldehyde *in vivo*, thereby inhibiting oxidative stress.

The ER is essential for maintaining hepatocyte metabolism and adapts to extracellular and intracellular stress signals (Lebeaupin et al., 2018). Upon prolonged ER stress, cells fail to maintain a normal ER function. Instead, pathways leading to an increase in ROS production induce cell death (Lebeaupin et al., 2018; Fernandes-da-Silva et al., 2021). Excessive alcohol consumption induces hyperhomocysteinemia, which interferes with protein folding and results in ER stress (Maiers and Malhi, 2019), and the ER stress-induced inflammation and apoptosis result in fibrosis (Liang et al., 2021). After the administration of KD, the expression of ER stress marker proteins was reduced, while the level of inflammation and the number of apoptotic cells also decreased.

Studies have indicated that the increase in AMPK phosphorylation alleviates liver injury (Hu et al., 2018; Wang et al., 2020). Hepatic autophagy alleviates liver injury by reducing cell apoptosis and inflammatory infiltration (Gilgenkrantz et al., 2021; Tong et al., 2021). These findings suggest that liver injury can be alleviated by activating AMPK and autophagy. Our results showed that KD treatment led to the activation of the AMPK signaling pathway by enhancing the expression of STRAD and LKB1, upstream of AMPK. Meanwhile, the activation of AMPK by silymarin did not involve LKB1, although both treatments reversed the inhibition of autophagy by ethanol.

Research shows that ALD is mainly caused by steatosis, oxidative stress, ER stress, and the toxic effects of acetaldehyde (Seitz et al., 2018; Maiers and Malhi, 2019). The diversity of the pathogenesis and the complexity of body functions allow drugs to play their roles *via* multiple mechanisms. Our research demonstrated that KD has a protective effect against alcoholic liver injury from oxidative stress, ER stress, and autophagy and has important preclinical value.

Although we found that KD alleviated alcoholic liver injury *via* multiple pathways, more studies are needed to clarify the specific target of KD. Subsequent studies should focus on ethanol metabolism, the AMPK signaling pathway, autophagy, oxidative stress, and ER stress to find the specific target of KD.

**FIGURE 9:**
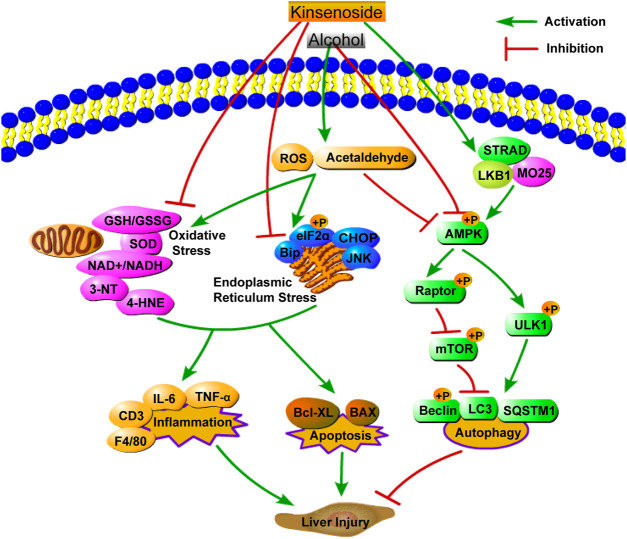
A simplified diagram of the mechanism of KD on alcoholic liver injury.

This study explored the pharmacological effects and mechanism of action of KD in alcoholic liver injury. A simplified diagram of the mechanism of KD action on alcoholic liver injury is shown in Figure 9. The results of this study suggested that KD can relieve inflammation and inhibit cell apoptosis by reducing oxidative stress and ER stress, while activating AMPK-dependent protective autophagy in mice with alcoholic liver injury. Currently, drugs for ALD treatment are limited and cannot meet the medication requirements of an increasing number of patients. Thus, together with the findings of previous studies, which have demonstrated the nontoxicity of KD *in vivo*, KD can be used as a supplement and candidate drug for the treatment of ALD.

## Data Availability

The original contributions presented in the study are publicly available. This data can be found here: Proteomics data are available *via* ProteomeXchange with identifier PXD027621.
