# Supplementary material for: Kinsenoside Alleviates Alcoholic Liver Injury by Reducing Oxidative Stress, Inhibiting Endoplasmic Reticulum Stress, and Regulating AMPK-Dependent Autophagy
Source: Front Pharmacol. 2022 Jan 18;12:747325. doi: 10.3389/fphar.2021.747325 (PMC8804359; doi:10.3389/fphar.2021.747325)
Supplement: Supplementary file 1 [file DataSheet1.docx]

Supplementary Material

**
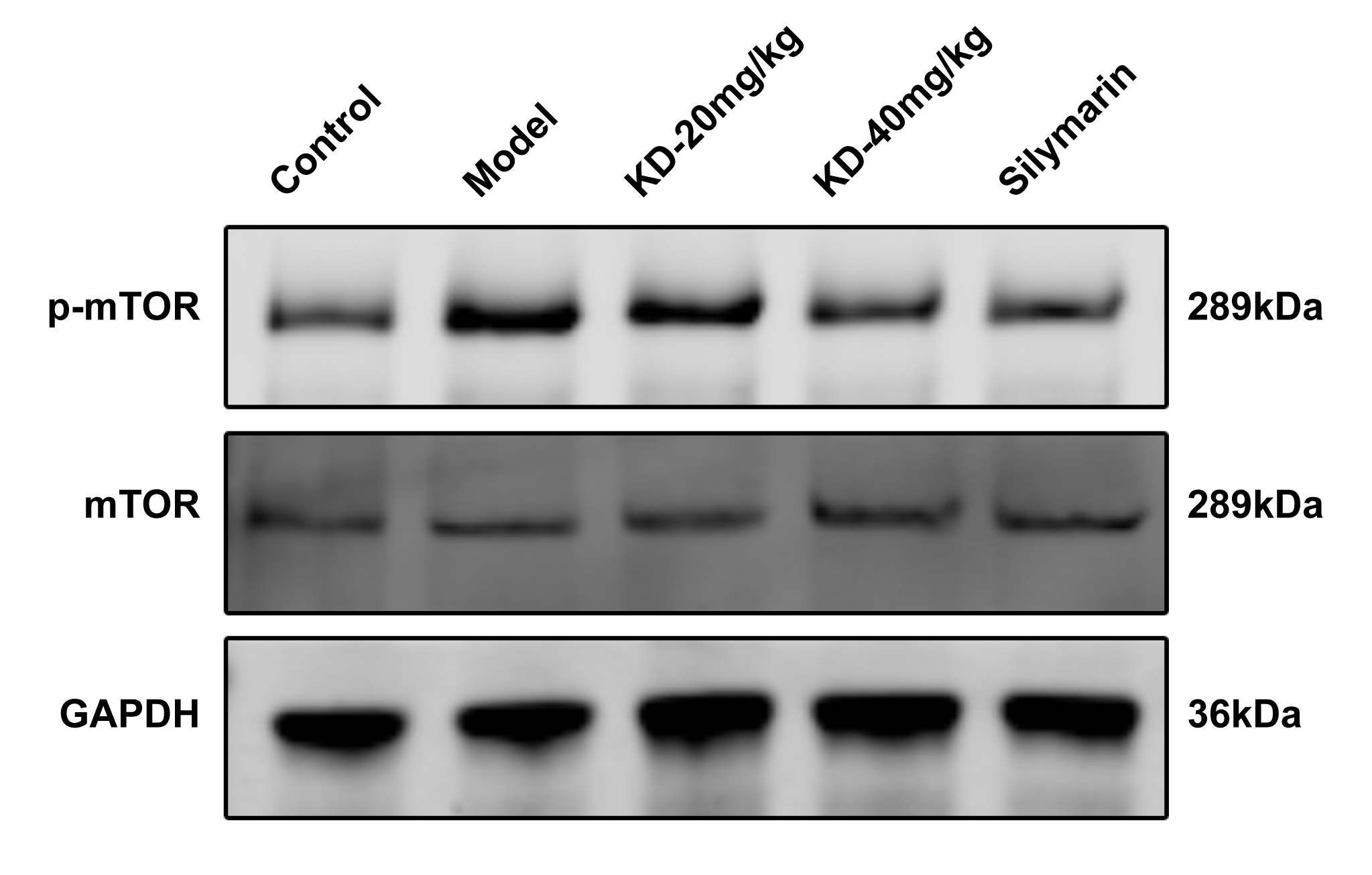
**

**Supplementary Figure 1.** Total mTOR strip and p-mTOR strip. Results showed that there was no change in the total mTOR protein expression level between the groups, while the phosphorylation level of mTOR in the model group increased, and the phosphorylation level decreased after KD treatment.

# Supplementary Method for Supplementary Figure 2

# Cell-Related Functional Damage Detection

# In order to study whether KD plays a key role in alcoholic liver injury by activating autophagy, we used autophagy inhibitors-chloroquine (CQ) to inhibit autophagy of AML12 to detect cell ALT/AST/TG levels. Cells were seeded in a 100mm culture dish at 4 × 10^6^ cells/dish. The next day, 50μM CQ or 40μΜ KD were pretreated for 1 hour, then followed by incubation with or without 200 mM ethanol for 24 hours, after which medium containing KD or CQ or ethanol was replaced. After another 24 hours, the ALT/AST/TG levels of the cells were detected according to the kit instructions.


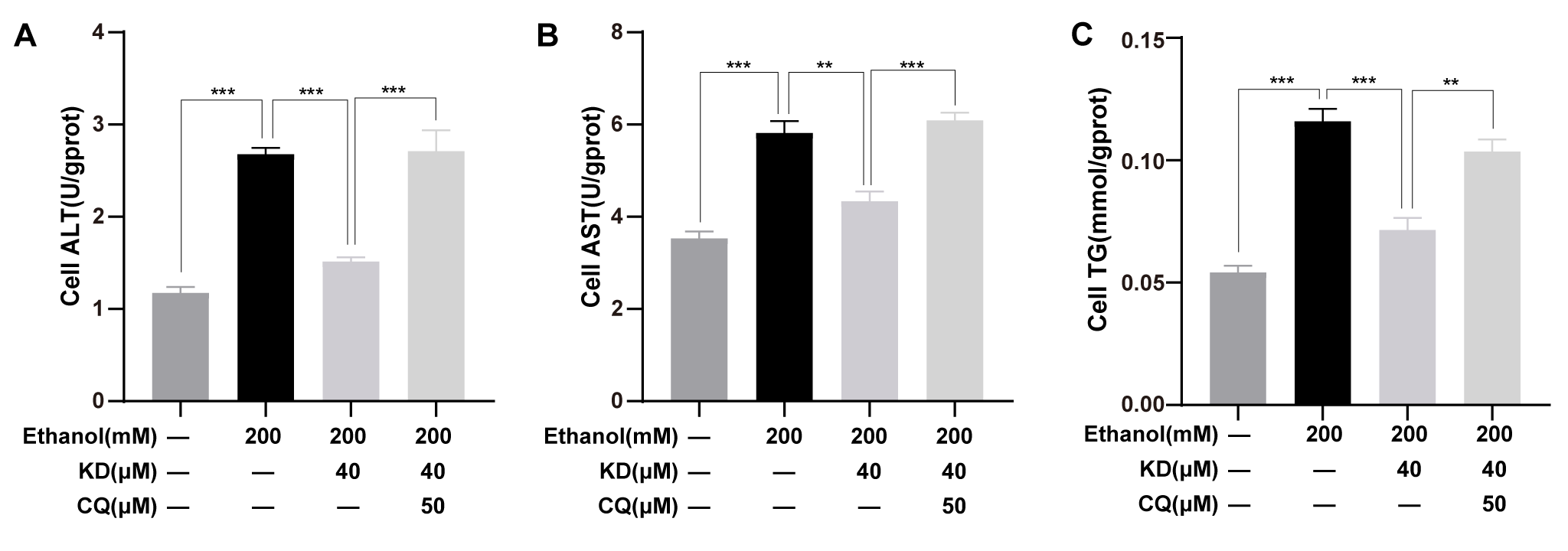


**Supplementary Figure 2.** After inhibiting autophagy, the protective effect of KD on alcoholic injury was abrogated. **(A)** ALT levels in AML12 cells. **(B)** AST levels in AML12 cells. **(C)** TG levels in AML12 cells. (n = 5). Data represent the mean ± SEM, **p* ＜ 0.05, ***p* ＜ 0.01, ****p* < 0.001.
